# Genome-Wide Identification and Transcriptional Expression Analysis of Cucumber Superoxide Dismutase (SOD) Family in Response to Various Abiotic Stresses

**DOI:** 10.1155/2017/7243973

**Published:** 2017-07-20

**Authors:** Yong Zhou, Lifang Hu, Hao Wu, Lunwei Jiang, Shiqiang Liu

**Affiliations:** ^1^School of Sciences, Jiangxi Agricultural University, Nanchang, Jiangxi, China; ^2^School of Agriculture, Jiangxi Agricultural University, Nanchang, Jiangxi, China; ^3^Key Laboratory of Crop Physiology, Ecology and Genetic Breeding, Ministry of Education, Jiangxi Agricultural University, Nanchang, Jiangxi, China; ^4^National Key Laboratory of Crop Genetic Improvement and National Centre of Plant Gene Research, Huazhong Agricultural University, Wuhan, China

## Abstract

Superoxide dismutase (SOD) proteins are widely present in the plant kingdom and play important roles in different biological processes. However, little is known about the *SOD* genes in cucumber. In this study, night *SOD* genes were identified from cucumber (*Cucumis sativus*) using bioinformatics-based methods, including 5 *Cu/ZnSOD*s, 3 *FeSODs*, and 1 *MnSOD*. Gene structure and motif analysis indicated that most of the *SOD* genes have relatively conserved exon/intron arrangement and motif composition. Phylogenetic analyses with SODs from cucumber and several other species revealed that these SOD proteins can be traced back to two ancestral SODs before the divergence of monocot and dicot plants. Many *cis*-elements related to stress responses and plant hormones were found in the promoter sequence of each *CsSOD* gene. Gene expression analysis revealed that most of the *CsSOD* genes are expressed in almost all the tested tissues. qRT-PCR analysis of 8 selected *CsSOD* genes showed that these genes could respond to heat, cold, osmotic, and salt stresses. Our results provide a basis for further functional research on *SOD* gene family in cucumber and facilitate their potential applications in the genetic improvement of cucumber.

## 1. Introduction

Under natural conditions, plants are frequently exposed to various stresses such as drought, salt, extreme temperature, and heavy metals, which may seriously affect their growth and development [1]. These stresses are surely accompanied by the generation of reactive oxygen species (ROS), including superoxide anion radicals (O_2_^−^), hydrogen peroxide (H_2_O_2_), hydroxyl radical (OH^−^), peroxyl radical (HOO^−^), and singlet oxygen (^1^O_2_), causing peroxidation and degradation of macromolecules and damage to cell membranes and ultimately leading to the death of cells [2–4]. In plant cells, ROS production is strictly controlled by enzymatic and nonenzymatic antioxidant defense systems including superoxide dismutase (SOD), catalase (CAT), ascorbate peroxidase (APX), monodehydroascorbate reductase (MDHAR), dehydroascorbate reductase (DHAR), thioredoxin, and glutathione. Among the antioxidant enzymes, superoxide dismutase (SOD, EC 1.15.1.1) can serve as an efficient scavenger of ROS by catalyzing the decomposition of superoxide anion radicals (O_2_^−^) to hydrogen peroxide (H_2_O_2_), which is further converted to nontoxic water and oxygen.

According to the binding metal cofactor(s) that interact with the active site, SODs can be classified into four types, including iron (FeSOD), manganese (MnSOD), copper/zinc (Cu/ZnSOD), and nickel (NiSOD) [4–6]. Different SOD isoforms have similar functions, but have different metal cofactors and amino acid sequences, crystal structures, and subcellular localizations, and exhibit different sensitivities to H_2_O_2_ in vitro [5, 7]. For example, KCN and H_2_O_2_ can inactivate Cu/ZnSODs and FeSODs irreversibly, but MnSOD is not sensitive to either of the chemicals [8]. Cu/ZnSODs, which are mainly located in chloroplasts, cytoplasm, and/or the extracellular space, are present in some bacteria and all eukaryotic species [9, 10], whereas MnSODs are mainly found in plant mitochondria [11]. At least one copy of MnSODs present in plant genomes plays a role in the scavenging of ROS in mitochondria [12]. FeSODs are distributed in prokaryotes, protozoans, and chloroplasts and cytoplasm of plants [13], while NiSODs were shown to be present in *Streptomyces* species, were predicted to exist in some cyanobacteria [14, 15], and have not been discovered in plants yet.

Many studies have shown that *SOD* gene transcripts are induced in various plant species under different forms of stress such as heat, cold, drought, and salt as well as osmotic and oxidative stresses and hormone signaling [5, 6, 9, 10, 16]. Additionally, different types of *SOD* genes exhibit different expression patterns under diverse stresses. In tomato, *SlSOD1* was the only gene that displayed markedly upregulated expression among the nine *SlSOD* genes, while the expression of four *SlSOD* genes (*SlSOD2*, *SlSOD5*, *SlSOD6*, and *SlSOD8*) was downregulated under salt treatment [6]. However, the four *SlSOD* genes (*SlSOD2*, *SlSOD5*, *SlSOD6*, and *SlSOD8*) showed increased expression levels under drought treatment [6]. Moreover, the same type of *SOD* genes do not share the same expression patterns under stresses. In banana, the transcript of *MaMSD1A* was increased under heat, drought, and salt stresses, and the expression of *MaMSD1B* was induced by heat and drought stresses, while the transcription of *MaMSD1C* was only induced by heat stress and *MaMSD1D* was not responsive to any stress [10]. Furthermore, some studies have revealed that the expression of some *SOD* genes can be regulated by alternative splicing [5, 10, 17, 18] and miRNAs [19, 20]. Certain genes were identified as positive regulators of abiotic stresses and can be one of the most important factors for plant's defense against various stresses. For example, transgenic plants overexpressing *SOD* genes displayed increased tolerance to a wide range of abiotic stresses including drought and salinity [21–26], oxidative stress [27], and stresses caused by the application of insecticides [28, 29].

Cucumber (*Cucumis sativus* L.), which belongs to the *Cucurbitaceae* family, is an economically and nutritionally important vegetable crop consumed worldwide [30]. *SOD* genes exist as a superfamily; however, no genome-wide information is available for *SOD* gene family in cucumber. In this study, we performed genome-wide identification of *SOD* gene family in cucumber and comprehensively analyzed their phylogenetic relationships, genome distribution, gene structure arrangement, conserved motifs, expression profiles in different tissues, and their expression patterns in response to various abiotic stresses, aiming to lay the foundation for functional characterization of them under various stresses.

## 2. Materials and Methods

### 2.1. Identification and Annotation of *SOD* Genes in Cucumber

A cucumber Cu/ZnSOD (GenBank number: XP_011648784.1) was used as a query sequence for tBLASTn against the cucumber genome from Cucumber Genome Initiative (CuGI, http://cucumber.genomics.org.cn/), which was released by the Institute of Vegetables and Flowers, Chinese Academy of Agricultural Sciences (IVF-CAAS). A name search for superoxide dismutase also helped to identify *SOD* genes in the NCBI database. The candidate SOD members were aligned to each other to guarantee that no member was analyzed for multiple times. To further confirm the accuracy of these SOD sequences, the cDNA sequences were translated into amino acid sequences, which were submitted to NCBI for domain prediction to eliminate the sequences without any SOD superfamily conserved domains. Subsequently, the conserved domains of these amino acid sequences were searched using the Simple Modular Architecture Research Tool (SMART) (http://smart.embl-heidelberg.de/) and Pfam (http://www.sanger.ac.uk/software/pfam/) online tools. The molecular weight and isoelectric point (pI) of each SOD protein were analyzed using the online ProtParam program (http://web.expasy.org/protparam/).

### 2.2. Subcellular Localization and Conserved Motif Prediction

Subcellular localization and signal peptide of SOD proteins were predicted using ProtComp 9.0 (http://linux1.softberry.com/berry.phtml/) and SignalP 4.1 (http://www.cbs.dtu.dk/services/SignalP/), respectively. Conserved motifs of SOD proteins were detected using the MEME program (http://meme-suite.org/tools/meme).

### 2.3. Multiple Sequence Alignments and Phylogenetic Analyses

Multiple sequence alignments of the deduced amino acid sequences of SOD proteins were performed using the ClustalW software with default parameters and then redrawn with the software Geneious Pro 4.8.3 (http://www.geneious.com/). Neighbor-joining (NJ) phylogenetic tree was constructed with the aligned SOD protein sequences using MEGA 5.1 software with the following parameters: poisson correction, pairwise deletion, and bootstrap (1000 replicates).

### 2.4. Intron/Exon Structure and Genome Distribution

The genomic sequences of *SOD* genes and their coding sequences were downloaded from CuGI (http://cucumber.genomics.org.cn/). The intron distribution pattern and splicing phase of each *CsSOD* gene were analyzed using Gene Structure Display Server (GSDS, http://gsds.cbi.pku.edu.cn/). To obtain the chromosomal locations of the *CsSOD* genes, mapping of the distribution of *CsSOD* genes throughout cucumber genome was performed with the MapInspect tool as previously described [31].

### 2.5. Promoter Region Analysis

The 1.0 kb DNA sequences upstream of the start codons of each *CsSOD* gene were retrieved from CuGI. Subsequently, the *cis*-elements in the promoters of each *CsSOD* gene were predicted using the PlantCARE tool (http://bioinformatics.psb.ugent.be/webtools/plantcare/html/).

### 2.6. Plant Materials and Treatments

Cucumber (*Cucumis sativus* L. cv. Chinese long 9930) was used in this research. The cucumber plants were planted in the field of Jiangxi Agricultural University (Nanchang, China). To examine the transcript levels of *CsSOD* members in different tissues, root, stem, leaf, flower, and fruit were collected from cucumber and immediately frozen in liquid nitrogen for storage at −80°C until use.

To analyze *CsSOD* members under various abiotic stresses, 2-week-old cucumber seedlings were adequately watered and grown in a greenhouse under the conditions of 16 h light/8 h dark at 24/18°C before the induction of different stresses. For heat treatment, cucumber seedlings were transferred to 50°C growth chambers under normal light conditions. For drought treatment, cucumber seedlings were grown in liquid Murashige and Skoog (MS) medium containing 300 mM PEG-6000. Cold and salt experiments for cucumber seedlings were conducted as previously described [31]. Leaves were collected at 0, 3, 6, 12, and 24 h after treatments and immediately frozen in liquid nitrogen for storage at −80°C until use.

### 2.7. RNA Extraction, RT-PCR, and Quantitative Real-Time PCR (qRT-PCR)

Total RNA was extracted with Trizol reagent (Tiangen Biotech Co., Ltd. Beijing, China) according to the manufacturer's instructions. Synthesis of first-strand cDNA and RT-PCR was performed using the methods described previously [31]. qRT-PCR was conducted as previously described [32]. Three replicates were performed for the analysis of each gene. The cucumber *Actin* gene was used as an internal control. The primers used in gene expression analysis are listed in Table S1 available online at https://doi.org/10.1155/2017/7243973.

## 3. Results

### 3.1. Genome-Wide Characterization of *CsSODs* in Cucumber

A total of nine cucumber *SOD* genes were identified from CuGI and NCBI database. The candidate genes were translated into amino acid sequences and analyzed for the presence of a SOD domain by searching SMART and Pfam database. The results showed that the cucumber genome contained five Cu/ZnSODs (CsCSD1–5), three FeSODs (CsFSD1–3), and one MnSOD (CsMSD), respectively (Table 1). All the three putative CsFSDs contained the C-terminal iron/manganese SOD domain (PF02777), and CsFSD2 and CsFSD3 had the N-terminal iron/manganese SOD alpha-hairpin domain (PF00081) (Table S2). All *CsCSDs* contained the copper-zinc domain (PF00080), which is a typical characteristic of Cu/ZnSOD. CsCSD3 contained an additional heavy-metal-associated domain (PF00403). Both of the N-terminal iron/manganese SOD alpha-hairpin domain (PF00081) and C-terminal iron/manganese SOD domain (PF02777) were present in CsMSD. In addition, CsFSD2 contained a glyoxalase domain (PF00903), which belongs to dioxygenase superfamily (Table S2). The three domains (PF00080, PF00081, and PF02777) were predicted to possess molecular function of oxidation-reduction process (GO:0055114) and superoxide metabolic process (GO:0006801) according to Gene Ontology [33], indicating that the putative CsSOD members belong to the SOD family.

These *CsSODs* were chosen for further analyses, and their characteristics are listed in Table 1. The gDNA lengths of *CsSODs* from the start to stop codons varied between 2056 and 4108 bp. Physicochemical analysis revealed that the lengths of putative CsSOD proteins varied between 152 and 377 amino acids. Besides, the putative CsSODs had a calculated molecular mass ranging from 15.3 to 42.5 kDa and a theoretical pI from 4.97 to 8.77 (Table 1). According to previous studies, all Cu/ZnSODs are acidic in character, while Fe-MnSODs are basic or acidic in character [6, 34–36]. In the present study, most of the CsSODs were acidic in character, except for three CsSODs (CsFSD1, CsMSD, and CsCSD4), which were basic in character.

Subcellular localizations of SOD proteins were predicted using ProtComp 9.0. The results showed that most of the CsCSDs (CsCSD1, CsCSD2, CsCSD3, and CsCSD4) were localized in the cytoplasm, while CsCSD5 was localized in the chloroplast (Table 1). In addition, two members (CsFSD1 and CsFSD2) and one member (CsFSD3) of FeSODs were located in the chloroplast and extracellular space, respectively. It seemed that SOD proteins with close phylogenetic relationships tended to have the same patterns of subcellular localization. The only member in group III, CsMSD, which is an MnSOD, was predicted to be localized in the mitochondrion. The prediction of the signal peptide by SignalP 4.1 indicated that only CsFSD3 had a signal peptide sequence with 16 amino acid residues that belongs to a secretory protein, confirming that CsFSD3 is an extracellular protein (Figure S1).

### 3.2. Conserved Motifs and Clustering Analysis of CsSODs

**Figure 1:**
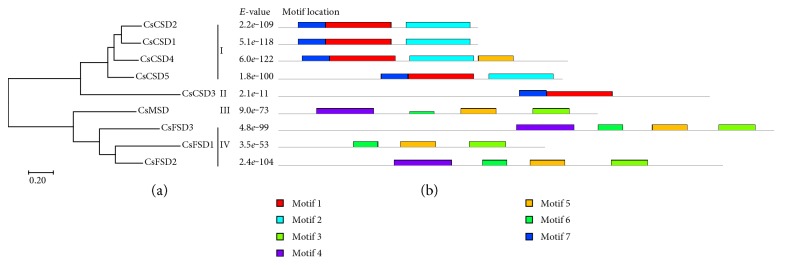
Unrooted neighbor-joining phylogenetic tree and conserved motif analysis of CsSOD proteins. (a) Phylogenetic tree was generated based on the protein sequences of CsSOD proteins. (b) Conserved motif analysis of CsSOD proteins. Different color boxes represent different types of motifs.

To investigate the evolutionary relationship of CsSODs in cucumber, an unrooted phylogenetic tree was created by aligning the 9 CsSOD protein sequences. CsSODs could be clustered into four groups (I, II, III, and IV), which was highly consistent with the types of their metal cofactors (Figure 1(a)).

We also conducted prediction of conserved motifs for CsSODs by MEME server. As shown in Figure 1(b), 7 motifs (motif 1 to motif 7) were identified. As expected, the SOD members in the same group of phylogenetic tree demonstrated very similar patterns of conserved motif distribution. Among them, the copper-zinc SOD domain (motifs 1, 2, and 7) was widely present in CSDs (group I: CsCSD1, CsCSD2, CsCSD4, and CsCSD5), except for CsCSD3 (group II), which included motifs 1 and 7. Three CsFSDs (CsFSD1, CsFSD2, and CsFSD3) composed group IV, which shared the iron/manganese SOD domain (motifs 3, 5, and 6) with CsMSD (group III). Motif 4 was related to iron/manganese SOD alpha-hairpin domain, which was located in group III (CsMSD) and group IV (CsFSD2 and CsFSD3, except for CsFSD1).

### 3.3. Multiple Sequence Alignment of CsSOD Proteins

To further explore the evolutionary relationships among cucumber SOD proteins, pairwise identities were calculated (Table S3). The results revealed that the similarity between CsSOD proteins was notably variable, and CsCSD proteins shared 27–72% identity at amino acid levels, while the identity of CsMSD and CsFSD proteins was 32–43%, which was also in accordance with the types of their metal cofactors.

**Figure 2:**
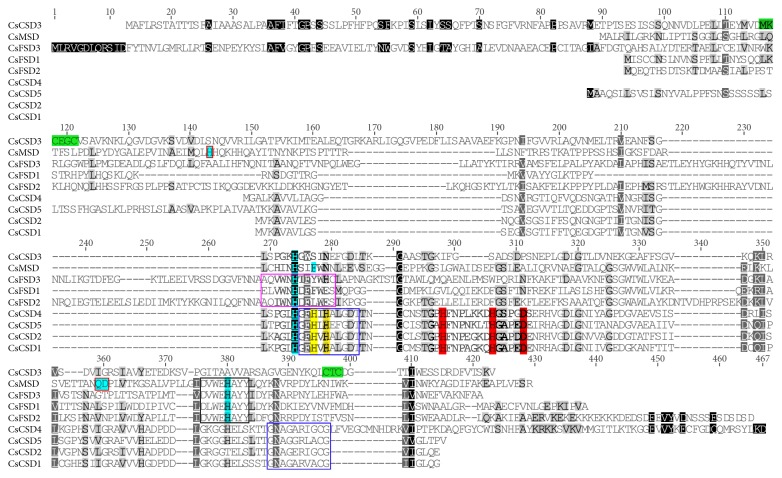
Multiple sequence alignment of deduced amino acid sequences of CsSOD proteins. Cu/ZnSOD signatures (GFH[VLI]H[EA][FL]GDTT and GNAG[GAE]R[VLI]ACG) are boxed in blue. The metal-binding sites for Cu^2+^ and Zn^2+^ are highlighted with yellow and red, respectively. The conserved metal-binding domain (DVWEHAYY) for Fe-MnSOD is in black box. The signature ([AE][QL][VI]WNH[TD]F[YFL]W[EH][CS]) responsible for the recognition of iron ion by FeSODs is boxed in pink. The conserved metal-binding motifs (MxCxxC and CxC) of CsCSD3 are highlighted in green. Six metal-binding sites in MnSOD are marked with cyan, and three residues (His, Gln, and Asp) that are present only in MnSOD and absent in FeSODs are marked with cyan in red box.

Multiple sequence alignment of the 9 CsSOD amino acid sequences was performed using the ClustalW software. The results showed that nearly all of the CsCSDs (except for CsCSD3) contained the Cu/ZnSOD signatures (GFH[VLI]H[EA][FL]GDTT and GNAG[GAE]R[VLI]ACG) and the metal-binding sites for Cu^2+^ and Zn^2+^ (Figure 2()). CsMSD along with CsFSDs had the conserved metal-binding domain “DVWEHAYY” with a conserved His residue found in almost all CsSODs. Three residues (His, Gln, and Asp) were present in CsMSD but absent in CsFSDs [10, 22], suggesting that these residues are specific for Mn^2+^. In addition, the signature ([AE][QL][VI]WNH[TD]F[YFL]W[EH][CS]) was responsible for the recognition of iron ion by FeSOD, which was also reported in previous studies [10, 37]. CsCSD3 was predicted to be located in extracellular space, suggesting that it participates in the secretory pathway. A blastp search in the NCBI database showed that CsCSD3 was 74.0% and 70.1% identical to copper chaperone for superoxide dismutase (CCS) proteins from rice (CuZn-SOD-CCh) [38] and banana (MaCCS) [39], respectively, implying that it belongs to the CCS subfamily. A conserved metal-binding motif (MxCxxC) and another conserved metal-binding motif (CxC) were near the N-terminus and in the C-terminus, respectively (Figure 2()).

### 3.4. Chromosomal Location and Gene Structure of *CsSODs*

**Figure 3:**
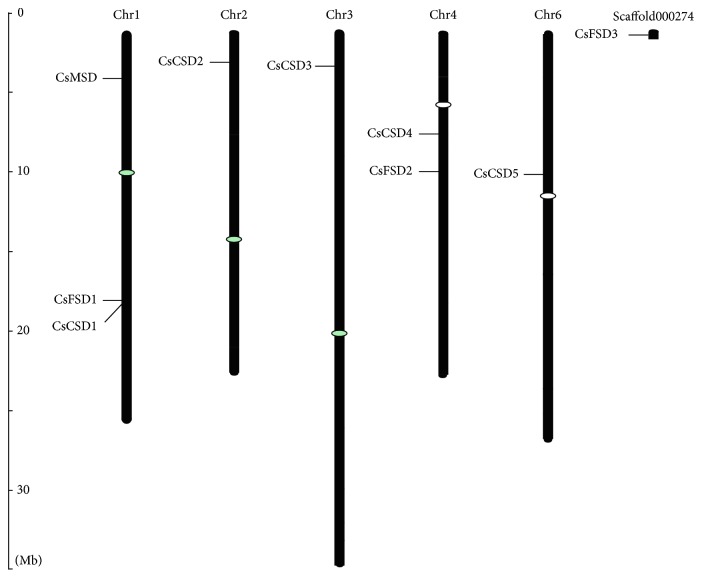
Chromosomal locations of 9 *CsSOD* genes on chromosomes of cucumber. The chromosome numbers are indicated at the top of chromosomes, and the size of chromosome is represented with a vertical scale.

The chromosomal distributions of cucumber *SOD* genes are shown in Figure 3(). Accordingly, the 9 *CsSODs* were distributed on 5 chromosomes in *Cucumis sativus*, except for *CsFSD3*, which was found on an unassembled sequence scaffold000274 (Figure 3()). The largest number of *SOD* genes was determined on Chr1 with 3 genes, while Chr4 possessed two *SOD* genes and all the others contained only one.

**Figure 4:**
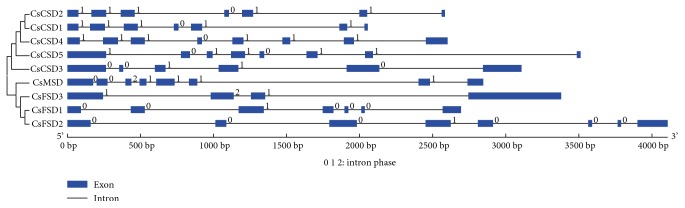
Exon-intron structures of *CsSOD* genes based on the phylogenetic relationship. Exons and introns are shown as green boxes and thin lines, respectively. Intron phases 0, 1, and 2 are indicated by numbers 0, 1, and 2.

To further examine the structural features of *CsSODs*, a comparison of the ORF sequences with the corresponding gDNA sequences was performed by GSDS server. As shown in Figure 4(), all *CsSOD* genes contained introns in their genomic sequences in cucumber, and the intron numbers in the *CsSOD* genes ranged from 3 to 7. Four *CsSOD* members (*CsCSD4*, *CsCSD5*, *CsMSD*, and *CsFSD2*) contained the largest number of introns (7 introns), while the smallest number was found in *CsFSD3* (3 introns). It is worth noting that two pairs of *CsSOD* genes (*CsCSD1* and *CsCSD2*, *CsFSD1* and *CsFSD2*) exhibited similar intron/exon organization patterns, respectively.

### 3.5. Phylogenetic Analysis of SOD Proteins in Plants

**Figure 5:**
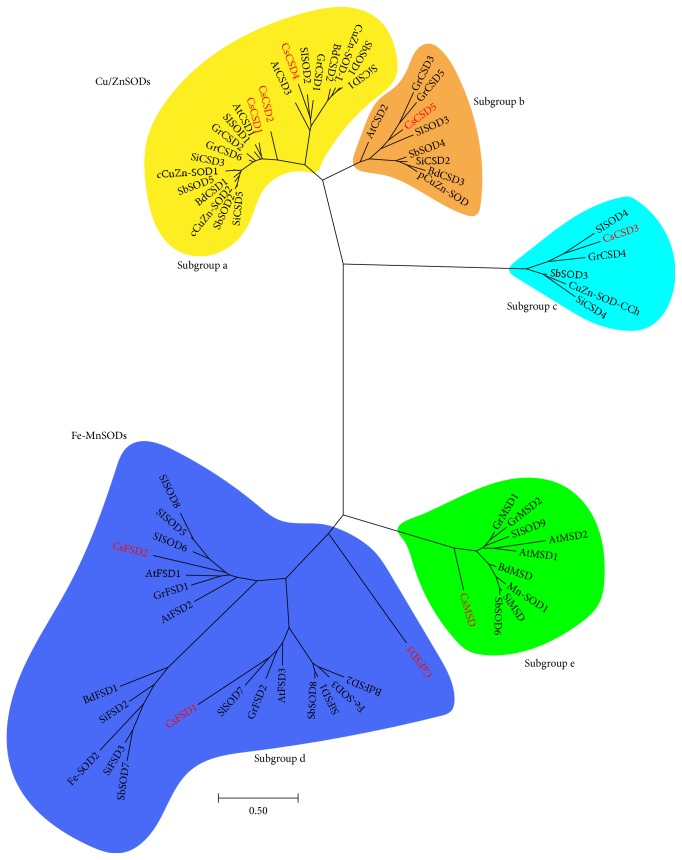
Phylogenetic tree of SOD proteins from cucumber and other plants. Deduced protein sequences of SOD proteins from different plant species were analyzed by ClustalW and then redrawn with the Geneious Pro software. Five colors (yellow, orange, cyan, blue, and green) represent five subgroups (a, b, c, d, and e), respectively. CsSOD proteins were highlighted in red. The amino acid sequences used to build the phylogenic tree were listed in Table S4.

To further investigate the phylogenetic relationships of SOD proteins between cucumber and other plant species, an unrooted neighbor-joining phylogenetic tree was generated using the amino acid sequences of the SOD proteins from *Arabidopsis thaliana*, *Brachypodium distachyon*, *Solanum lycopersicum*, *Oryza sativa*, *Sorghum bicolor*, *Gossypium raimondii*, and *Setaria italica*. The phylogenetic tree revealed that these SOD proteins could be classified into two major groups: Cu/ZnSODs and Fe-MnSODs. Cu/ZnSODs included three subgroups (a–c), while Fe-MnSODs were divided into two subgroups (d and e) (Figure 5), implying that FeSODs and MnSODs originated from a common ancestor [11].

Like in other plants, CsSODs were present in all the five subgroups. Three CsCSDs (CsCSD1, CsCSD2, and CsCSD4) along with SbSOD1, SlSOD1, and other cytoplasmic Cu/ZnSODs were clustered into subgroup a, while CsCSD5 together with SbSOD4, SlSOD3, and other chloroplastic Cu/ZnSODs was clustered in another cluster (subgroup b). The remaining CsCSDs (CsCSD3), along with SlSOD4, SbSOD3, GrCSD4, and other CSDs, were clustered in subgroup c, which was like a junction between Cu/ZnSODs and Mn-FeSODs clusters. Notably, all of the three CsFSDs were clustered in subgroup d with other plant FeSODs (Figure 5()). Among them, CsFSD1 and CsFSD2 were clustered with other plant chloroplastic FeSODs, while CsFSD3, which was found on an unassembled sequence and predicted to be located in extracellular space (Table 1; Figure 3()), was separated from other plant FeSODs. In addition, CsCSDs and CsFSDs (except for CsFSD3) were clustered into Cu/ZnSODs and FeSODs of dicot-specific branches, respectively, revealing the presence of two ancestral SODs before the divergence of monocot and dicot plants. Moreover, CsMSD together with other plant mitochondrial MSDs were clustered in subgroup e (Figure 5()).

### 3.6. Analysis of *cis*-Acting Regulatory Elements in the Promoter of *CsSODs*

To understand the gene function and regulation patterns of *CsSODs*, sequences of 1000 bp upstream regions from the translation start site of each *CsSOD* were identified from CuGI and were subsequently determined using PlantCARE. The results showed that 59 types of *cis*-acting regulatory elements were found in the promoter region of *CsSODs* and all *CsSOD* promoters contained typical TATA and CAAT boxes, which are essential elements of the promoters (Table S5). In addition, several *cis*-elements related to developmental processes such as circadian control (circadian), flavonoid biosynthetic gene regulation (MBSII), meristem expression (CAT-box) and zein metabolism regulation (O2-site), and tissue-specific expression, including shoot-specific element (as-2-box), meristem specific activation element (CCGTCC-box), and endosperm-specific elements (GCN4 and Skn-1 motif), were present in *CsSOD* promoters. Among them, the light-responsive elements included 23 different types and were the largest group of elements. Each *CsSOD* promoter contained 3–7 types of light-responsive elements, implying that *CsSODs* might be differentially regulated by light. The type numbers of the remaining *cis*-acting regulatory elements, including stress-related and hormone-related elements, were shown in Table 2. The *cis*-acting regulatory elements involved in abiotic stress, such as heat stress-responsive element (HSE), drought-responsive element (MBS), wound-responsive element (WUN motif), defense and stress-responsive element (TC-rich repeats), anaerobic induction element (ARE), and elicitor-responsive elements (EIRE and Box-W1), were also detected in a series of members. Moreover, 8 types of hormone-responsive *cis*-elements were identified, and all of the *CsSOD* promoter sequences contained at least one type of *cis*-elements involved in plant hormone response, including abscisic acid- (ABA-) responsive element (ABRE), methyl jasmonate- (MeJA-) responsive element (CGTCA-motif), ethylene-responsive element (ERE), gibberellin-responsive elements (GARE-motif, P-box, and TATC-box), salicylic acid-responsive elements (TCA-element), and auxin-responsive elements (TGA-element). Numerous *cis*-elements related to light, stress, or hormone responses were present in the promoter of *CsSODs*, suggesting that *CsSODs* are related to the responses to various stresses and hormones.

### 3.7. Expression Profiles of *CsSODs* in Different Tissues

**Figure 6:**
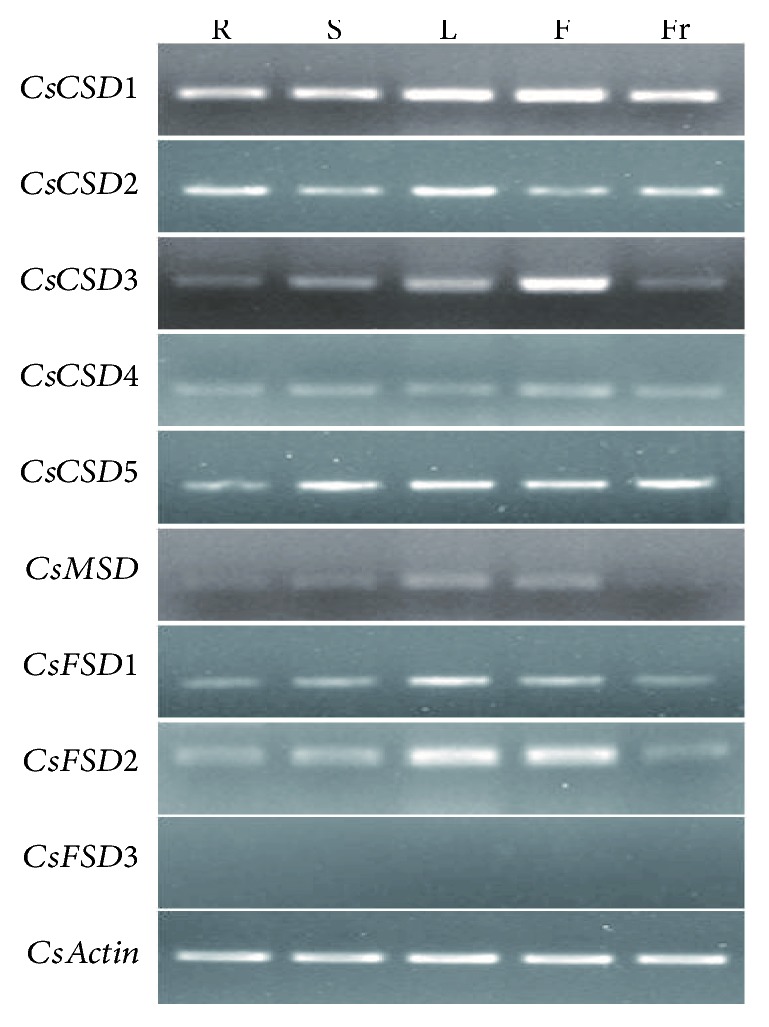
RT-PCR analysis of cucumber *SOD* genes in different tissues. RNA was isolated from different tissues, including root (R), stem (S), leaf (L), flower (F), and fruit (Fr).

To investigate the functions of *CsSODs*, their expression profiles in different tissues (root, stem, leaf, flower, and fruit) were determined by RT-PCR. The results showed that most of the *CsSODs* were expressed in almost all 5 tissues, except for *CsMSD*, which was not expressed in fruit, and *CsFSD3*, whose transcript was not detected in all the tested tissues (Figure 6). In addition, *CsCSD1* was consistently highly expressed in all 5 tissues, whereas *CsCSD4* displayed an extremely low expression level. *CsCSD1* was more highly expressed in leaf and flower than in other tested tissues. The expression of *CsCSD2* in root and leaf was significantly higher than that in other tested tissues, while that of *CsCSD5* in root was significantly lower than that in other tested tissues. Unlike other *CsCSDs*, *CsCSD3* displayed a distinctly tissue-specific expression pattern and had the highest expression level in flower. *CsFSD1* and *CsFSD2*, both of which were FeSODs (Table 1), showed similar expression patterns (Figure 6). *CsFSD1* and *CsFSD2* showed varied expression levels across tissues and were abundantly expressed in leaf and flower, but moderately expressed in other tissues (Figure 6). The expression of *CsMSD* was relatively lower in leaf, extremely low in flower and stem, and even not detected in root and fruit.

### 3.8. Expression of *CsSODs* in Response to Various Abiotic Stresses

**Figure 7:**
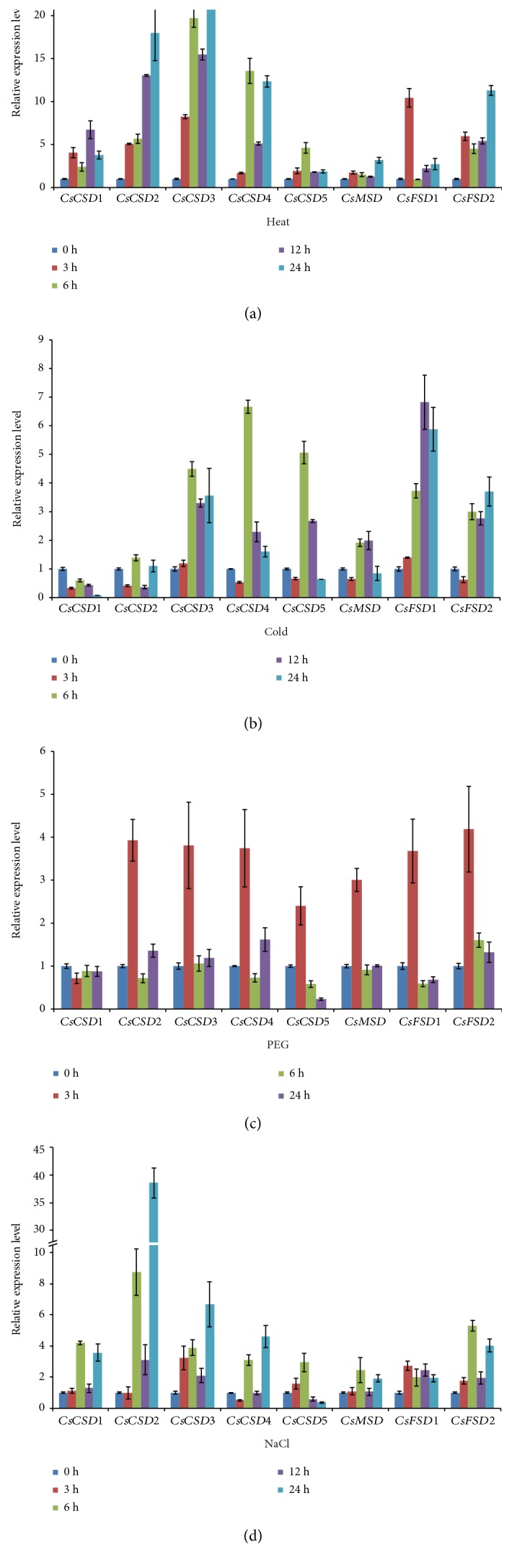
qRT-PCR analysis of cucumber *SOD* genes in response to various abiotic stresses including heat (a), cold (b), PEG (c), and NaCl (d). Error bars indicate SD based on three biological replicates.

To investigate the roles of *CsSOD* genes in response to various abiotic stresses, we analyzed the expression patterns of *CsSOD* genes under heat, cold, drought, and NaCl treatments by qRT-PCR. As shown in Figure 7, the expression of *CsSOD* genes was noticeably different under different abiotic stress treatments, and their expression patterns were complex. Exceptionally, no expression of *CsFSD3* was detected in response to these stresses. In addition, the expression of *CsFSD3* was not detected in root, stem, leaf, flower, and fruit (Figure 6). These results implied that *CsFSD3* is a pseudogene.

All of the remaining 8 *CsSOD* genes were strongly induced by heat treatment and exhibited more than 3- to 24-fold increase in expression. Among them, 1 gene (*CsFSD1*), 2 genes (*CsCSD4*, *CsCSD5*), 1 gene (*CsCSD1*), and 4 genes (*CsCSD2*, *CsCSD3*, *CsMSD*, and *CsFSD2*) reached the highest expression levels at 3 h, 6 h, 12 h, and 24 h, respectively. Interestingly, the expression of a pair of *CSDs*, *CsCSD3* and *CsCSD4*, whose transcripts were induced gradually until 6 h, subsequently decreased at 12 h and finally reached a higher level at 24 h (Figure 7(a)). In addition, the expression of *CsFSD1* was increased sharply, reached the highest level at 3 h, then dropped dramatically at 6 h, and subsequently increased gradually as the treatment continued (Figure 7(a)). Under cold treatment, the expression levels of most *CsSOD* genes decreased at 3 h (*CsCSD3* remained constant, and *CsFSD1* was upregulated slightly) and then increased markedly at 6 h (Figure 7(b)). After 6 h, all of the *CsCSD* genes were downregulated sharply at 12 h and continued to decline at 24 h (except for *CsCSD2*, whose expression was increased at 24 h). After 6 h, the expression of *CsFSD1* and *CsFSD2* was induced gradually, and the induction peaked at 12 h and 24 h, respectively. During PEG treatment, nearly all *CsCSD* genes were strongly induced, their expression peaked at 3 h, and then the transcript levels were decreased, whereas *CsCSD1* was slightly downregulated at 3 h and its transcript remained constant (Figure 7(c)). Under NaCl treatment, the expression of most *CsSOD* genes was induced gradually until 6 h, then declined at 12 h, and ultimately increased at 24 h (Figure 7(d)). It was worth noting that the transcript of *CsCSD2* reached the highest level (38.6-fold) at 24 h, which was obviously higher than that of other *CsSOD* genes. The expression of *CsCSD5* increased gradually until 6 h, then declined gradually after 12 h of treatment, and finally decreased dramatically at 24 h (Figure 7(d)).

## 4. Discussion

### 4.1. *SOD* Genes in Cucumber and Their Evolution

SOD plays important roles in multiple processes of plant growth and resistance against environment stresses, and *SOD* gene family has been reported to be widely distributed in different plant species, such as Arabidopsis [40], longan [41], rice [34, 42], poplar [22], banana [10], sorghum [35], tomato [6], and cotton [36, 43]. However, no comprehensive analysis of this gene family has been reported in cucumber. In the present study, a total of 9 *SOD* genes were identified in cucumber, which cover all three major types of plant *SOD* genes, including 5 Cu/ZnSODs, 3 FeSODs, and 1 MnSOD (Table 1). Similar results were obtained in other plant species. For example, previous studies revealed that the numbers of *SOD* genes in Arabidopsis, rice, sorghum, and tomato are 8 (3 Cu/ZnSODs, 2 MnSODs, and 3 FeSODs), 8 (5 Cu/ZnSODs, 1 MnSOD, and 2 FeSODs), 8 (5 Cu/ZnSODs, 1 MnSOD, and 2 FeSODs), and 9 (4 Cu/ZnSODs, 1 MnSOD, and 4 FeSODs), respectively. There are large differences in genome size, and the number of *SOD* genes varies among these plant species, but does not vary proportionally along with the changes in genome size. The discrepancy in the number of *SOD* genes in different plant species may be attributed to gene duplication, which consists of tandem and segmental duplications and plays a crucial role in the expansion of *SOD* genes for diversification. Gene duplication of *SOD* genes was also found in different plant species [35, 36, 43]. In our study, no segmental duplication event was observed between these genes according to the criteria of a previous study [35]. Therefore, tandem duplication most likely plays an important role in the expansion of *CsSOD* genes, just like the two adjacent genes *CsFSD1* and *CsCSD1* in Chr1 (Figure 3()).

Gene structure analysis revealed that the intron numbers of the 9 cucumber *SOD* genes were 3–7 (Figure 4()). A previous research reported that plant *SOD* genes have highly conserved intron patterns, and most cytosolic and chloroplastic SODs harbor 7 introns [44]. In our study, four members (*CsCSD4*, *CsCSD5*, *CsMSD*, and *CsFSD2*) were predicted to contain 7 introns (Figure 4()). The divergence of *CsSOD* gene structure may be due to the mechanisms including exon/intron gain/loss, exonization/pseudoexonization, and insertion/deletion according to a previous study [45], and the SOD members in the same clade of phylogenetic tree displayed similar exon-intron organization patterns (such as *CsCSD1* and *CsCSD2*, *CsFSD1* and *CsFSD2*), suggesting that they may have similar functions related to various abiotic stresses.

Phylogenetic analysis of SOD proteins between cucumber and 7 other plant species showed that the SODs could be divided into two groups of Cu/ZnSODs and Fe-MnSODs, and these two groups could be further divided into 5 subgroups with robust statistical support, which is consistent with the results of previous reports [6, 10, 11, 35]. Most of the subcellular localization data of SODs supported the phylogenetic data. For example, several pairs of Cu/ZnSODs, including CsCSD1 and CsCSD2, SbSOD1 and SbSOD2 [35], SlSOD1 and SlSOD2 [6], cCuZn-SOD-1 and cCuZn-SOD-2 [42], GrCSD1 and GrCSD2 [36], were predicted to be located in the cytoplasm in subgroup a (Figure 5()). Chloroplastic Cu/ZnSODs, chloroplastic FeSODs, and mitochondrial MnSODs were clustered into subgroups b, d, and e, respectively. CsCSD3, along with CuZn-SOD-CCh and other chloroplastic CSDs, was observed in subgroup c, suggesting that it is a member of plant CCS family. In addition, almost all CsSODs were phylogenetically clustered with at least one member from all the considered plant species (Figure 5()), suggesting that CsSODs probably have the same functions as SODs in other plant species. However, CsFSD3, which was found on an unassembled sequence and predicted to be located in extracellular space (Figures 1() and 3()), was separated from other plant FeSODs. Besides, the prediction of the signal peptide revealed that CsFSD3 has a signal peptide sequence belonging to a secretory protein (Figure S1), implying that CsFSD3 is an extracellular protein.

### 4.2. Expression Patterns of *CsSODs* in Different Tissues

Comprehensive gene expression analyses of *SOD* family genes have demonstrated that *SODs* have diverse expression patterns in different tissues of plants, such as Arabidopsis [40], rice [34, 42], poplar [22], banana [10], tomato [6], and cotton [36, 43]. In this study, the results of RT-PCR analysis showed that most of the *CsSOD* genes are ubiquitously expressed and a few genes display tissue-specific expression patterns except for *CsFSD3*, whose transcript was not detected in any tested tissues (Figure 6). For instance, *CsCSD1* showed high expression in all the tested tissues, whereas *CsCSD4* displayed extremely low expression level (Figure 6), suggesting that they have differential expression patterns in various tissues. In addition, many *CsSOD* genes showed the highest expression in leaves, which is consistent with previous studies of other species [9, 36]. Notably, all of the *CsSOD* genes except for *CsMSD* were expressed in fruit (Figure 6) and shared a common *cis*-acting regulatory element Skn-1_motif (Table 2), which is required for endosperm expression, suggesting that these genes may be involved in fruit development. *CsCSD3* exhibited high expression in flower, indicating its specific roles required in flower development.

### 4.3. Functions of *CsSODs* in Response to Various Abiotic Stresses

Analysis of the *cis*-elements in *CsSOD* gene promoters resulted in the detection of three major types of *cis*-elements associated with light, abiotic stress, and hormone response as well as *cis*-elements related to developmental processes and tissue-specific expression (Table S5). A relatively large number of light-responsive *cis*-elements were detected in *CsSOD* gene promoters, suggesting that *CsSODs* might participate in light response. Some studies have shown that *SOD* genes are involved in the response to light in different plants including *Arabidopsis thaliana* [19, 40], *Nicotiana tabacum* [46], and *Nicotiana plumbaginifolia* [47]. Transgenic plants overexpressing a pea chloroplastic Cu/ZnSOD showed increased resistance to high light [48]. In addition, the *cis*-acting regulatory element involved in circadian control was found in six *CsSODs* except for *CsCSD4*, *CsCSD5*, and *CsMSD* (Table S5). Therefore, CsSODs, especially chloroplastic SODs, may be responsible for light response and function in the efficient removal of the superoxide formed during photosynthetic electron transport and under various stress conditions in the light.

In addition, a series of *cis*-elements related to abiotic stress responses were identified in *CsSOD* gene promoters, such as HSE, MBS, WUN-motif, TC-rich repeats, ARE, EIRE, and Box-W1 (Table 2), which may regulate gene expression under various stresses. Most of the *SOD* genes in Arabidopsis, tomato, and banana can be induced in response to various abiotic stresses such as heat, cold, drought, and salinity [6, 10, 40] and are considered to participate in the elimination of ROS caused by various abiotic stresses [11, 40]. In the present study, the expression of *CsFSD3* was not detected under various abiotic stresses (Figure 7), and its transcripts were also undetectable in different tissues (Figure 6), indicating that it is a pseudogene. The expression levels of other 8 *CsSOD* genes were largely altered under stress conditions, suggesting that these genes play important regulatory roles in stress response and that there may be functional redundancy. For example, in this study, all of the detected *CsSOD* genes were upregulated under heat treatment, and some of them displayed similar expression patterns (Figure 7(a)). Under cold treatment, the expression levels of all *CsSOD* genes were obviously altered and their expression patterns were distinct from each other (Figure 7(b)), implying that *CsSOD* genes may have different functions in response to cold stress. Among them, the expression of *CsCSD1* was decreased gradually, with an expression pattern similar to that of *MaCSD1B* and *MaCSD2B* [10]. For PEG treatment, nearly all of the *CsSOD* genes had similar expression patterns, which reached the highest levels at 3 h and then decreased (Figure 7(c)), implying that the functions of *CsSOD* genes are related to osmotic stress. During NaCl treatment, although the transcripts of most *CsSOD* genes were changed, *CsCSD2* was the only gene that showed a remarkable increase among the 8 *CsSOD* genes (Figure 7(d)), demonstrating that *CsCSD2* might play a predominant antioxidant role under salt stress. This phenomenon was previously observed for *SlSOD1* in tomato [6]. Both SlSOD1 and CsCSD2 were clustered in subgroup a and showed a relatively high amino acid sequence identity (72.8%) (Figure 5). Taken together, we propose that the *CsSOD* genes have different functions in ROS elimination caused by different abiotic stresses, and may play vital roles in adversity adaptation of plants. In addition, some *CsSOD* genes were found to respond to various abiotic stresses with different expression patterns. For example, *CsCSD1* was downregulated by cold treatment and upregulated by heat and NaCl treatments, while its expression remained constant without a significant change under PEG stress (Figure 7), implying a divergence in the functions of *CsCSD1* in different signal pathways. Further studies are needed to reveal the exact functions of *CsSOD* genes during various abiotic stresses in cucumber.

## 5. Conclusion

In the present study, we identified 9 putative *SOD* genes from cucumber and analyzed their classification, genome organization, gene structure, conserved motif, and phylogenetic relationships. Subsequently, RT-PCR and qRT-PCR were carried out to analyze the expression profiles of *CsSOD* genes in different tissues and under various abiotic stresses including heat, cold, PEG, and NaCl. It could be suggested that *CsSOD* genes play vital roles in almost all tissues at the stage of growth and development in cucumber. This study provides a comprehensive understanding and facilitates further functional characterization of the *SOD* gene family in cucumber and lays a foundation for elucidating the molecular mechanisms of *SOD* genes during stress response and development of plants.

## Supplementary Material

Fig. S1. The signal peptide prediction of the CsSOD proteins. The purple line indicate the discrimination score which is used to discriminate signal peptides from non-signal peptides. Table S1. Primers used in this study. Table S2. A list of predicted domains in 9 CsSODs in our study. Table S3. Pairwise amino acid sequence comparison of CsSOD proteins. Table S4. SOD sequences used for phylogenetic tree analysis. Table S5. The predicted promoter elements of CsSOD genes.

## Figures and Tables

**Table 1:** A complete list of 9 *CsSODs* identified in this study.

Isoforms	Gene ID	Chromosamal location	CuGI (5′–3′)	gDNA size (bp)	Intron number	Length (aa)	Theoretical Mw (kDa)	pI	ProtComp
CsCSD1	Csa001740	1	17723177–17725232	2056	6	152	15.3	5.44	Cytoplasm
CsCSD2	Csa006777	2	1729871–1727288	2584	6	152	15.4	4.97	Cytoplasm
CsCSD3	Csa018002	3	2262114–2259007	3108	5	328	34.8	5.16	Cytoplasm
CsCSD4	Csa017461	4	6531097–6533698	2602	7	220	23.3	8.77	Cytoplasm
CsCSD5	Csa015219	6	8963271–8966782	3512	7	216	21.9	5.87	Chloroplast
CsMSD	Csa004428	1	2960859–2958014	2846	6	243	27.0	8.54	Mitochondrion
CsFSD1	Csa001483	1	17559641–17556948	2694	6	203	23.4	8.29	Chloroplast
CsFSD2	Csa016620	4	9018790–9014683	4108	8	338	38.6	5.75	Chloroplast
CsFSD3	Csa022092	Scaffold000274	12020–15398	3379	3	377	42.5	5.16	Extracellular

**Table 2:** Kinds and numbers of known stress-related and hormone-related *cis*-elements in the promoter regions of *CsSOD* genes.

	Stress-related *cis*-element	Hormone-related *cis*-element
*CsCSD1*	TC-rich repeats (1), ARE (2)	ABRE (3), TCA-element (1), TGA-element (1)
*CsCSD2*	MBS (3), WUN-motif (1), TC-rich repeats (1), ARE (1)	GARE-motif (2), TCA-element (1)
*CsCSD3*	HSE (3), TC-rich repeats (3), ARE (1)	ABRE (1), CGTCA-motif (1), GARE-motif (2), TATC-box (1), TCA-element (1)
*CsCSD4*	HSE (3), MBS (3), ARE (1)	ABRE (1), CGTCA-motif (1), ERE (3), TCA-element (1)
*CsCSD5*	MBS (3), TC-rich repeats (1), ARE (1)	TATC-box (1), TGA-element (1)
*CsMSD*	HSE (1), MBS (1), TC-rich repeats (2), ARE (1), Box-W1 (1)	ABRE (1), ERE (1), GARE-motif (1), TGA-element (1)
*CsFSD1*	HSE (2), MBS (2), TC-rich repeats (1)	GARE-motif (1)
*CsFSD2*	HSE (1)	ABRE (3), P-box (2), TCA-element (2)
*CsFSD3*	HSE (2), MBS (1), EIRE (1)	ABRE (1), CGTCA-motif (1)

The numbers in parentheses indicate the number of the *cis*-element in the promoter of *CsSOD*.

## References

[1] Cramer G. R., Urano K., Delrot S., Pezzotti M., Shinozaki K. (2011). Effects of abiotic stress on plants: a systems biology perspective. *BMC Plant Biology*.

[2] You J., Chan Z. (2015). ROS regulation during abiotic stress responses in crop plants. *Frontiers in Plant Science*.

[3] Del Rio L. A. (2015). ROS and RNS in plant physiology: an overview. *Journal of Experimental Botany*.

[4] Mittler R., Vanderauwera S., Gollery M., Van Breusegem F. (2004). Reactive oxygen gene network of plants. *Trends in Plant Science*.

[5] Yan J. J., Zhang L., Wang R. Q. (2016). The sequence characteristics and expression models reveal superoxide dismutase involved in cold response and fruiting body development in *Volvariella volvacea*. *International Journal of Molecular Sciences*.

[6] Feng K., Yu J., Cheng Y. (2016). The *SOD* gene family in tomato: identification, phylogenetic relationships, and expression patterns. *Frontiers in Plant Science*.

[7] Abreu I. A., Cabelli D. E. (2010). Superoxide dismutases-a review of the metal-associated mechanistic variations. *Biochimica et Biophysica Acta (BBA) - Proteins and Proteomics*.

[8] Asada K., Yoshikawa K., Takahashi M., Maeda Y., Enmanji K. (1975). Superoxide dismutases from a blue-green alga, *Plectonema boryanum*. *Journal of Biological Chemistry*.

[9] Pilon M., Ravet K., Tapken W. (2011). The biogenesis and physiological function of chloroplast superoxide dismutases. *Biochimica et Biophysica Acta (BBA) - Bioenergetics*.

[10] Feng X., Lai Z., Lin Y., Lai G., Lian C. (2015). Genome-wide identification and characterization of the superoxide dismutase gene family in *Musa acuminata* cv. Tianbaojiao (AAA group). *BMC Genomics*.

[11] Alscher R. G., Erturk N., Heath L. S. (2002). Role of superoxide dismutases (SODs) in controlling oxidative stress in plants. *Journal of Experimental Botany*.

[12] Moller I. M. (2001). Plant mitochondria and oxidative stress: electron transport, NADPH turnover, and metabolism of reactive oxygen species. *Annual Review of Plant Physiology and Plant Molecular Biolog*.

[13] Miller A. F. (2012). Superoxide dismutases: ancient enzymes and new insights. *FEBS Letters*.

[14] Wuerges J., Lee J. W., Yim Y. I., Yim H. S., Kang S. O., Djinovic Carugo K. (2004). Crystal structure of nickel-containing superoxide dismutase reveals another type of active site. *Proceedings of the National Academy of Sciences of the United States of America*.

[15] Youn H. D., Kim E. J., Roe J. H., Hah Y. C., Kang S. O. (1996). A novel nickel-containing superoxide dismutase from *Streptomyces* spp. *Biochemical Journal*.

[16] Wang B., Luttge U., Ratajczak R. (2004). Specific regulation of SOD isoforms by NaCl and osmotic stress in leaves of the C_3_ halophyte *Suaeda salsa* L.. *Journal of Plant Physiology*.

[17] Srivastava V., Srivastava M. K., Chibani K. (2009). Alternative splicing studies of the reactive oxygen species gene network in Populus reveal two isoforms of high-isoelectric-point superoxide dismutase. *Plant Physiology*.

[18] Feng W., Hongbin W., Bing L., Jinfa W. (2006). Cloning and characterization of a novel splicing isoform of the iron-superoxide dismutase gene in rice (*Oryza sativa* L.). *Plant Cell Reports*.

[19] Sunkar R., Kapoor A., Zhu J. K. (2006). Posttranscriptional induction of two Cu/Zn superoxide dismutase genes in Arabidopsis is mediated by downregulation of miR398 and important for oxidative stress tolerance. *Plant Cell*.

[20] Dugas D. V., Bartel B. (2008). Sucrose induction of Arabidopsis miR398 represses two Cu/Zn superoxide dismutases. *Plant Molecular Biology*.

[21] Lee S. H., Ahsan N., Lee K. W. (2007). Simultaneous overexpression of both CuZn superoxide dismutase and ascorbate peroxidase in transgenic tall fescue plants confers increased tolerance to a wide range of abiotic stresses. *Journal of Plant Physiology*.

[22] Molina-Rueda J. J., Tsai C. J., Kirby E. G. (2013). The Populus superoxide dismutase gene family and its responses to drought stress in transgenic poplar overexpressing a pine cytosolic glutamine synthetase (GS1a). *PLoS One*.

[23] Wang F. Z., Wang Q. B., Kwon S. Y., Kwak S. S., Su W. A. (2005). Enhanced drought tolerance of transgenic rice plants expressing a pea manganese superoxide dismutase. *Journal of Plant Physiology*.

[24] Wang M., Zhao X., Xiao Z., Yin X., Xing T., Xia G. (2016). A wheat superoxide dismutase gene *TaSOD2* enhances salt resistance through modulating redox homeostasis by promoting NADPH oxidase activity. *Plant Molecular Biology*.

[25] Jing X., Hou P., Lu Y. (2015). Overexpression of copper/zinc superoxide dismutase from mangrove *Kandelia candel* in tobacco enhances salinity tolerance by the reduction of reactive oxygen species in chloroplast. *Frontiers in Plant Science*.

[26] Negi N. P., Shrivastava D. C., Sharma V., Sarin N. B. (2015). Overexpression of CuZnSOD from *Arachis hypogaea* alleviates salinity and drought stress in tobacco. *Plant Cell Reports*.

[27] Kim Y. H., Lim S., Han S. H. (2015). Expression of both CuZnSOD and APX in chloroplasts enhances tolerance to sulfur dioxide in transgenic sweet potato plants. *Comptes Rendus Biologies*.

[28] Perl A., Perl-Treves R., Galili S. (1993). Enhanced oxidative-stress defense in transgenic potato expressing tomato Cu, Zn superoxide dismutases. *Theoretical and Applied Genetics*.

[29] Luo X., Wu J., Li Y. (2013). Synergistic effects of *GhSOD1* and *GhCAT1* overexpression in cotton chloroplasts on enhancing tolerance to methyl viologen and salt stresses. *PLoS One*.

[30] Hu L., Liu S. (2012). Genome-wide analysis of the MADS-box gene family in cucumber. *Genome*.

[31] Hu L., Yang Y., Jiang L., Liu S. (2016). The *catalase* gene family in cucumber: genome-wide identification and organization. *Genetics and Molecular Biology*.

[32] Zhou Y., Liu L., Huang W. (2014). Overexpression of *OsSWEET5* in rice causes growth retardation and precocious senescence. *PLoS One*.

[33] Gotz S., Garcia-Gomez J. M., Terol J. (2008). High-throughput functional annotation and data mining with the Blast2GO suite. *Nucleic Acids Research*.

[34] Dehury B., Sarma K., Sarmah R. (2013). *In silico* analyses of superoxide dismutases (SODs) of rice (*Oryza sativa* L.). *Journal of Plant Biochemistry and Biotechnology*.

[35] Filiz E., Tombuloğlu H. (2015). Genome-wide distribution of superoxide dismutase (SOD) gene families in *Sorghum bicolor*. *Turkish Journal of Biology*.

[36] Zhang J., Li B., Yang Y. (2016). Genome-wide characterization and expression profiles of the superoxide dismutase gene family in Gossypium. *International Journal of Genomics*.

[37] Parker M. W., Blake C. C. F. (1988). Iron- and manganese-containing superoxide dismutases can be distinguished by analysis of their primary structures. *FEBS Letters*.

[38] Huang C. H., Kuo W. Y., Weiss C., Jinn T. L. (2012). Copper chaperone-dependent and -independent activation of three copper-zinc superoxide dismutase homologs localized in different cellular compartments in Arabidopsis. *Plant Physiology*.

[39] Feng X., Chen F., Liu W. (2016). Molecular characterization of *MaCCS*, a novel copper chaperone gene involved in abiotic and hormonal stress responses in *Musa acuminata* cv. Tianbaojiao. *International Journal of Molecular Sciences*.

[40] Kliebenstein D. J., Monde R. A., Last R. L. (1998). Superoxide dismutase in *Arabidopsis*: an eclectic enzyme family with disparate regulation and protein localization. *Plant Physiology*.

[41] Lin Y. L., Lai Z. X. (2013). Superoxide dismutase multigene family in longan somatic embryos: a comparison of CuZn-SOD, Fe-SOD, and Mn-SOD gene structure, splicing, phylogeny, and expression. *Molecular Breeding*.

[42] Nath K., Kumar S., Poudyal R. S. (2014). Developmental stage-dependent differential gene expression of superoxide dismutase isoenzymes and their localization and physical interaction network in rice (*Oryza sativa* L.). *Genes & Genomics*.

[43] Wang W., Xia M., Chen J. (2016). Genome-wide analysis of superoxide dismutase gene family in *Gossypium raimondii* and *G. arboreum*. *Plant Gene*.

[44] Fink R. C., Scandalios J. G. (2002). Molecular evolution and structure―function relationships of the superoxide dismutase gene families in angiosperms and their relationship to other eukaryotic and prokaryotic superoxide dismutases. *Archives of Biochemistry and Biophysics*.

[45] Xu G., Guo C., Shan H., Kong H. (2012). Divergence of duplicate genes in exon-intron structure. *Proceedings of the National Academy of Sciences of the United States of America*.

[46] Kurepa J., Montagu M. V., Inzé D. (1997). Expression of *sodCp* and *sodB* genes in *Nicotiana tabacum*: effects of light and copper excess. *Journal of Experimental Botany*.

[47] Tsang E. W., Bowler C., Herouart D. (1991). Differential regulation of superoxide dismutases in plants exposed to environmental stress. *Plant Cell*.

[48] Gupta A. S., Heinen J. L., Holaday A. S., Burke J. J., Allen R. D. (1993). Increased resistance to oxidative stress in transgenic plants that overexpress chloroplastic Cu/Zn superoxide dismutase. *Proceedings of the National Academy of Sciences of the United States of America*.

