# Supplementary figures and images for: Genome-Wide Identification and Transcriptional Expression Analysis of Cucumber Superoxide Dismutase (SOD) Family in Response to Various Abiotic Stresses
Source: Int J Genomics. 2017 Jul 20;2017:7243973. doi: 10.1155/2017/7243973 (PMC5541821; doi:10.1155/2017/7243973)

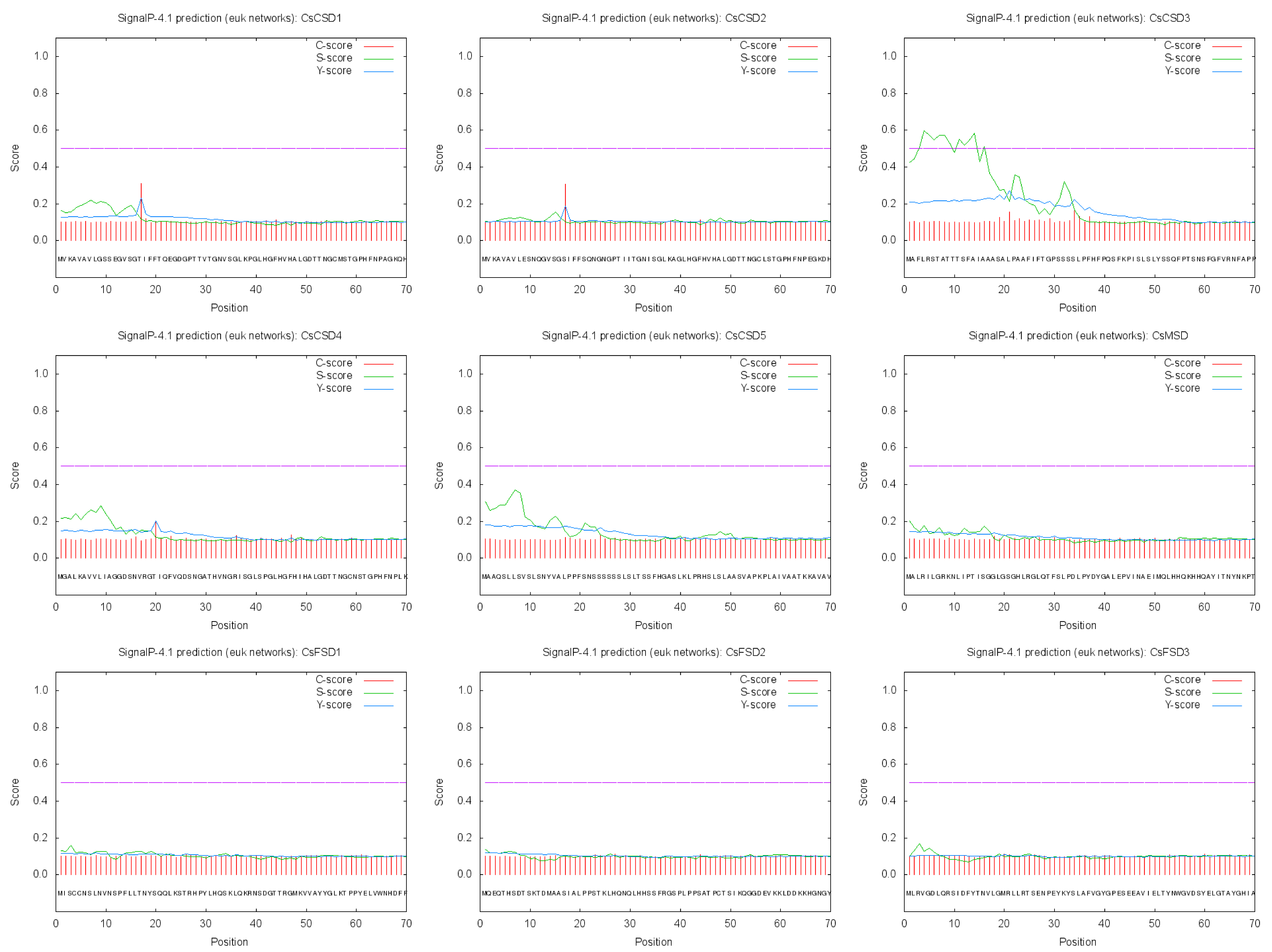

Supplement: Supplementary file 2 [file 7243973.f2.doc]
