# Supplementary material for: Genome-Wide Identification and Transcriptional Expression Analysis of Cucumber Superoxide Dismutase (SOD) Family in Response to Various Abiotic Stresses
Source: Int J Genomics. 2017 Jul 20;2017:7243973. doi: 10.1155/2017/7243973 (PMC5541821; doi:10.1155/2017/7243973)
Supplement: Supplementary file 3 [file 7243973.f3.doc]

**Table S1.** Primers used in this study.

| **Gene** | **Forward primer** | **Reverse primer** |
| --- | --- | --- |
| RT-PCR | | |
| CsCSD1 | ACGCCTTAGGTGACACAACC | ATGAACCACAACAGCCCTTC |
| CsCSD2 | AATGTGGTTGCTGGTGATGA | CCAATAACGCCACAGCCTAT |
| CsCSD3 | GGAAAGCATGGTTGGTCAAT | GGCACTGATTTGTCTTCGGT |
| CsCSD4 | GGGCACGAACTTAGCAAGAC | CCACCCTTGGTCTTCAGTGT |
| CsCSD5 | GGGTGACCTGGGAAACATAA | AGAATAGGGGCCGCTAAGAG |
| CsMSD | GAAGAGGTTTCAGAAGGGGG | CCCAAGCAGAGGCACTAAAG |
| CsFSD1 | GGGTTTGGCTTGTTTTGAAG | TTCACGAAACACTCTGCTCG |
| CsFSD2 | CGTTTGGGAGCATGCTTATT | TCGTCGCTATCTTCGTCCTT |
| CsFSD3 | CCTTCTACTGGCACTGCCTC | CAGCCTCTTTTACCAGCCAG |
| CsActin | GACATTCAATGTGCCTGCTATG | CATACCGATGAGAGATGGCTG |
| qRT-PCR | | |
| CsCSD1 | CCACTGTTACGGGTAATGTT | GGAGCACCATGTTGTTTT |
| CsCSD2 | GGGAGTCAGTGGAAGCAT | TGTGGCCCAGTTGATAAG |
| CsCSD3 | TAATGAGCCTCTGGGTGA | ACTCGCAGCTTCTGTTTG |
| CsCSD4 | ATTTCGGATAGGCTGATTT | CATTTCCAGTTGTCTTGCT |
| CsCSD5 | GCACATTTCAATCCTAACAA | TGCCTCTGCTACTCCATC |
| CsMSD | GCTCTGAATAAAGAACTGAAGAA | AAACATCAATCCCAAGCA |
| CsFSD1 | AACTCTCCATTTCTGCTAACC | CGACCTTCATTCCTCTTGT |
| CsFSD2 | AAGCCTCCTCCATATCCTC | CTGTGATGTTTTCCCCAA |
| CsFSD3 | GCCGATCTTCAATCTCTCTT | AATAACCCTTCCCACAGC |
| CsActin | GAATCCAGCACGATACCA | TCAACCCAAAGGCTAACA |
