# Supplementary material for: Genome-Wide Identification and Transcriptional Expression Analysis of Cucumber Superoxide Dismutase (SOD) Family in Response to Various Abiotic Stresses
Source: Int J Genomics. 2017 Jul 20;2017:7243973. doi: 10.1155/2017/7243973 (PMC5541821; doi:10.1155/2017/7243973)
Supplement: Supplementary file 4 [file 7243973.f4.doc]

**Table S2.** A list of predicted domains in 9 CsSODs in our study.

| **Isoforms** | **Alignment (start-end)** | **envelope (start-end)** | **hmm acc** | **hmm name** | **E-value** |
| --- | --- | --- | --- | --- | --- |
| CsFSD1 | 85**–**182 | 85**–**182 | PF02777 | iron/manganese SOD domain | 2.1e-32 |
| CsCSD1 | 12**–**148 | 8**–**148 | PF00080 | copper-zinc SOD domain | 2.5e-47 |
| CsMSD | 29**–**117 | 29**–**118 | PF00081 | iron/manganese SOD alpha-hairpin domain | 6.2e-21 |
|  | 128**–**229 | 128**–**231 | PF02777 | iron/manganese SOD domain | 1.2e-35 |
| CsCSD2 | 11**–**148 | 8**–**148 | PF00080 | copper-zinc SOD domain | 2.4e-47 |
| CsCSD3 | 180**–**285 | 176**–**306 | PF00080 | copper-zinc SOD domain | 4.3e-17 |
|  | 99**–**156 | 101**–**155 | PF00403 | Heavy-metal-associated domain | 1.2e-10 |
| CsFSD2 | 88**–**172 | 88**–**173 | PF00081 | iron/manganese SOD alpha-hairpin domain | 4.3e-27 |
|  | 180**–**291 | 180**–**293 | PF02777 | iron/manganese SOD domain | 1.4e-35 |
| CsCSD4 | 14**–**149 | 10**–**151 | PF00080 | copper-zinc SOD domain | 1.0e-46 |
| CsCSD5 | 74**–**211 | 71**–**211 | PF00080 | copper-zinc SOD domain | 7.3e-45 |
| CsFSD3 | 181**–**261 | 181**–**261 | PF00081 | iron/manganese SOD alpha-hairpin domain | 1.3e-34 |
|  | 281**–**373 | 269**–**374 | PF02777 | iron/manganese SOD domain | 2.2e-33 |
|  | 2**–**92 | 1**–**102 | PF00903 | Glyoxalase | 3.4e-11 |
