# Supplementary material for: Genome-Wide Identification and Transcriptional Expression Analysis of Cucumber Superoxide Dismutase (SOD) Family in Response to Various Abiotic Stresses
Source: Int J Genomics. 2017 Jul 20;2017:7243973. doi: 10.1155/2017/7243973 (PMC5541821; doi:10.1155/2017/7243973)
Supplement: Supplementary file 5 [file 7243973.f5.doc]

**Table S3.** Pairwise amino acid sequence comparison of CsSOD proteins.

|  | CsCSD1 | CsCSD2 | CsCSD3 | CsCSD4 | CsCSD5 | CsMSD | CsFSD1 | CsFSD2 | CsFSD3 |
| --- | --- | --- | --- | --- | --- | --- | --- | --- | --- |
| CsCSD1 | 100 |  |  |  |  |  |  |  |  |
| CsCSD2 | 72 | 100 |  |  |  |  |  |  |  |
| CsCSD3 | 30 | 27 | 100 |  |  |  |  |  |  |
| CsCSD4 | 65 | 65 | 33 | 100 |  |  |  |  |  |
| CsCSD5 | 64 | 60 | 38 | 60 | 100 |  |  |  |  |
| CsMSD |  |  |  |  |  | 100 |  |  |  |
| CsFSD1 |  |  |  |  |  | 36 | 100 |  |  |
| CsFSD2 |  |  |  |  |  | 33 | 43 | 100 |  |
| CsFSD3 |  |  |  |  |  | 32 | 38 | 42 | 100 |

Values present percent sequence identity.
