# Supplementary material for: Genome-Wide Identification and Transcriptional Expression Analysis of Cucumber Superoxide Dismutase (SOD) Family in Response to Various Abiotic Stresses
Source: Int J Genomics. 2017 Jul 20;2017:7243973. doi: 10.1155/2017/7243973 (PMC5541821; doi:10.1155/2017/7243973)
Supplement: Supplementary file 6 [file 7243973.f6.doc]

**Table S4.** SOD sequences used for phylogenetic tree analysis.

| **Name** | **Protein ID** | **Species** | **Type** |
| --- | --- | --- | --- |
| CsCSD1 | Csa001740 | *Cucumis sativus* | Cu-Zn |
| CsCSD2 | Csa006777 | *Cucumis sativus* | Cu-Zn |
| CsCSD3 | Csa018002 | *Cucumis sativus* | Cu-Zn |
| CsCSD4 | Csa017461 | *Cucumis sativus* | Cu-Zn |
| CsCSD5 | Csa015219 | *Cucumis sativus* | Cu-Zn |
| CsMSD | Csa004428 | *Cucumis sativus* | Mn |
| CsFSD1 | Csa001483 | *Cucumis sativus* | Fe |
| CsFSD2 | Csa016620 | *Cucumis sativus* | Fe |
| CsFSD3 | Csa022092 | *Cucumis sativus* | Fe |
|  |  |  |  |
| SlSOD1 | Solyc01g067740.2 | *Solanum lycopersicum* | Cu-Zn |
| SlSOD2 | Solyc03g062890.2 | *Solanum lycopersicum* | Cu-Zn |
| SlSOD3 | Solyc11g066390.1 | *Solanum lycopersicum* | Cu-Zn |
| SlSOD4 | Solyc08g079830.2 | *Solanum lycopersicum* | Cu-Zn |
| SlSOD5 | Solyc06g048410.2 | *Solanum lycopersicum* | Fe |
| SlSOD6 | Solyc03g095180.2 | *Solanum lycopersicum* | Fe |
| SlSOD7 | Solyc02g021140.2 | *Solanum lycopersicum* | Fe |
| SlSOD8 | Solyc06g048420.1 | *Solanum lycopersicum* | Fe |
| SlSOD9 | Solyc06g049080.2 | *Solanum lycopersicum* | Mn |
|  |  |  |  |
| cCuZn-SOD1 | AAA33917.1 | *Oryza sativa* | Cu-Zn |
| cCuZn-SOD2 | BAA00800.1 | *Oryza sativa* | Cu-Zn |
| CuZn-SOD-L | LOC_Os03g11960 | *Oryza sativa* | Cu-Zn |
| pCuZn-SOD | BAA12745.1 | *Oryza sativa* | Cu-Zn |
| CuZn-SOD-CCh | LOC_Os04g48410 | *Oryza sativa* | Cu-Zn |
| Mn-SOD1 | AAA57130.1 | *Oryza sativa* | Mn |
| Fe-SOD3 | BAA37131.1 | *Oryza sativa* | Fe |
| Fe-SOD2 | BAG99354.1 | *Oryza sativa* | Fe |
|  |  |  |  |
| SbSOD1 | Sobic.001G453800.1 | *Sorghum bicolor* | Cu-Zn |
| SbSOD2 | Sobic.002G407900.3 | *Sorghum bicolor* | Cu-Zn |
| SbSOD3 | Sobic.006G185700.1 | *Sorghum bicolor* | Cu-Zn |
| SbSOD4 | Sobic.007G166600.1 | *Sorghum bicolor* | Cu-Zn |
| SbSOD5 | Sobic.001G371900.1 | *Sorghum bicolor* | Cu-Zn |
| SbSOD6 | Sobic.009G093200.1 | *Sorghum bicolor* | Mn |
| SbSOD7 | Sobic.010G012900.1 | *Sorghum bicolor* | Fe |
| SbSOD8 | Sobic.010G033000.1 | *Sorghum bicolor* | Fe |
|  |  |  |  |
| AtCSD1 | AT1G08830.1 | *Arabidopsis thaliana* | Cu-Zn |
| AtCSD2 | AT2G28190.1 | *Arabidopsis thaliana* | Cu-Zn |
| AtCSD3 | AT5G18100.1 | *Arabidopsis thaliana* | Cu-Zn |
| AtMSD1 | AT3G10920.1 | *Arabidopsis thaliana* | Mn |
| AtMSD2 | AT3G56350.1 | *Arabidopsis thaliana* | Mn |
| AtFSD1 | AT4G25100.1 | *Arabidopsis thaliana* | Fe |
| AtFSD2 | AT5G51100.1 | *Arabidopsis thaliana* | Fe |
| AtFSD3 | AT5G23310.1 | *Arabidopsis thaliana* | Fe |
|  |  |  |  |
| BdCSD1 | Bradi1g18340 | *Brachypodium distachyon* | Cu-Zn |
| BdCSD2 | Bradi1g69680 | *Brachypodium distachyon* | Cu-Zn |
| BdCSD3 | Bradi3g43070 | *Brachypodium distachyon* | Cu-Zn |
| BdMSD | Bradi2g30580 | *Brachypodium distachyon* | Mn |
| BdFSD1 | Bradi1g50550 | *Brachypodium distachyon* | Fe |
| BdFSD2 | Bradi1g51140 | *Brachypodium distachyon* | Fe |
|  |  |  |  |
| GrCSD1 | Gorai.007G261100.1 | *Gossypium raimondii* | Cu-Zn |
| GrCSD2 | Gorai.013G192900.6 | *Gossypium raimondii* | Cu-Zn |
| GrCSD3 | Gorai.006G104900.2 | *Gossypium raimondii* | Cu-Zn |
| GrCSD4 | Gorai.004G205500.1 | *Gossypium raimondii* | Cu-Zn |
| GrCSD5 | Gorai.009G090300.2 | *Gossypium raimondii* | Cu-Zn |
| GrCSD6 | Gorai.013G116800.4 | *Gossypium raimondii* | Cu-Zn |
| GrFSD1 | Gorai.001G052700.1 | *Gossypium raimondii* | Fe |
| GrFSD2 | Gorai.013G068400.1 | *Gossypium raimondii* | Fe |
| GrMSD1 | Gorai.009G293300.3 | *Gossypium raimondii* | Mn |
| GrMSD2 | Gorai.011G207900.2 | *Gossypium raimondii* | Mn |
|  |  |  |  |
| SiCSD1 | Seita.9G488200.1 | *Setaria italica* | Cu-Zn |
| SiCSD2 | Seita.6G251600.1 | *Setaria italica* | Cu-Zn |
| SiCSD3 | Seita.9G403600.1 | *Setaria italica* | Cu-Zn |
| SiCSD4 | Seita.7G206200.1 | *Setaria italica* | Cu-Zn |
| SiCSD5 | Seita.2G422500.1 | *Setaria italica* | Cu-Zn |
| SiMSD | Seita.3G286500.1 | *Setaria italica* | Mn |
| SiFSD1 | Seita.4G031200.1 | *Setaria italica* | Fe |
| SiFSD2 | Seita.4G011900.1 | *Setaria italica* | Fe |
| SiFSD3 | Seita.9G123900.1 | *Setaria italica* | Fe |
